# New persistent opioid use following robotic-assisted, laparoscopic and open surgery inguinal hernia repair

**DOI:** 10.1007/s00464-024-11040-1

**Published:** 2024-07-22

**Authors:** Ian T. MacQueen, Gediwon Milky, I.-Fan Shih, Feibi Zheng, David C. Chen

**Affiliations:** 1 Department of Surgery, David Geffen School of Medicine at UCLA, Los Angeles, CA USA; 2 Global Access, Value & Economics, Intuitive Surgical, Sunnyvale, CA USA; 3 DeBakey Department of Surgery, Baylor College of Medicine, Houston, TX USA; 4 UCLA Division of General Surgery, Los Angeles, USA; 5 Lichtenstein Amid Hernia Clinic at UCLA, Los Angeles, USA

**Keywords:** Persistent opioid use, Robotic surgery, Minimally invasive surgery, Inguinal hernia repair

## Abstract

**Introduction:**

Post-operative prescription opioid use is a known risk factor for persistent opioid use. Despite the increased utilization of robotic-assisted surgery (RAS) for inguinal hernia repair (IHR), little is known whether this minimally invasive approach results in less opioid consumption. In this study, we compare long-term opioid use between RAS versus laparoscopic (Lap) versus open surgery for IHR.

**Methods:**

A retrospective cohort study of opioid-naïve patients who underwent outpatient primary IHR was conducted using the Merative™ MarketScan® (Previously IBM MarketScan®) Databases between 2016 and 2020. Patients not continuously enrolled 180 days before/after surgery, who had malignancy, pre-existing chronic pain, opioid dependency, or invalid prescription fill information were excluded. Among patients exposed to opioids peri-operatively, we assessed long-term opioid use as any opioid prescription fill within 90 to 180 days post-surgery. Secondary outcomes were controlled substance schedule II/III opioid fill, and high-dose opioid fill defined as > 50 morphine milligram equivalent per day. An Inverse-probability of treatment weighted logistic regression was used to compare outcomes between groups with *p*-value of < 0.05 considered statistically significant.

**Results:**

A total of 41,271 patients were identified (2070 (5.0%) RAS, 16,704 (40.5%) Lap, and 22,497 (54.5%) open surgery). RAS was associated with less likelihood of prescription fills for any opioid (OR 0.78, 95% CI 0.60 to 0.98 versus Lap; OR 0.67, 95% CI 0.52 to 0.85 versus open), and schedule II/III opioid (OR 0.74, 95% CI 0.56 to 0.96 versus Lap; OR 0.68, 95% CI 0.51 to 0.88 versus open), but comparable high-dose opioid fill (OR 0.95, 95% CI 0.54 to 1.55 versus Lap; OR 0.96, 95% CI 0.56 to 1.52 versus open). Lap and open surgery had no significant difference.

**Conclusion:**

In this cohort of patients derived from a national commercial claims dataset, patients undergoing RAS had a decreased risk of long-term opioid use compared to laparoscopic and open surgery patients undergoing IHR.

**Supplementary Information:**

The online version contains supplementary material available at 10.1007/s00464-024-11040-1.

The opioid epidemic is devastating in its scope and its impact on the lives of those affected. Three million Americans are afflicted by opioid use disorder and drug overdoses now account for over 90,000 deaths annually in the United States [1, 2]. Mitigation strategies have included treatments for those affected and prevention efforts to avoid long-term opioid use and abuse. Exposure to opioid medication around the time of surgery is a clear risk factor for long-term opioid use [3]. It is incumbent upon surgeons to understand the risks specific to individual patients and operations in order to combat the rising prevalence of chronic opioid use and addiction.

Inguinal hernia repair is a very common procedure and is associated with more pain as compared to many outpatient operations. Over 800,000 inguinal hernia repairs has been estimated to happen every year in the United States [4]. Prior studies suggest that greater than 90% of inguinal hernia repair patients are discharged with an opioid prescription following surgery [5, 6]. It is unknown how many of these patients become long-term users of opioids, and what patient and operative factors impact the risk of progressing to long-term opioid use.

There are multiple methods and approaches for performing inguinal hernia repair including open techniques and minimally invasive techniques by laparoscopy (Lap) or robotic-assisted surgery (RAS). For mesh-based repair, the approach is not considered to significantly affect recurrence, and has little clear impact on the development of severe chronic-post operative pain [7, 8]. Clinical circumstances often dictate which approach is ideal, such as selecting open mesh repair for patients with high cardiac risk who wish to avoid general anesthesia or minimally invasive repair for patients with recurrence after prior open repair [8]. For the majority of patients, any of these options is reasonable and the recommendation is often based on perceived minor benefits of one technique over another or on surgeon preference. Minimally invasive preperitoneal mesh repair is commonly considered to cause less acute post-operative pain as compared to open mesh repair, though data on this are conflicting [8, 9]. Overall, patterns of pain and analgesic use after these operations are incompletely understood.

Prior studies have examined different approaches to repair and the association with opioid use in the short-term post-operative period [6, 9, 10]. Other studies have examined an association between prescribing patterns and amount of opioid use post operatively [5, 11]. Although these outcomes are crucial in understanding the health impacts and financial costs of these operations in the short-term, any link between these short-term outcomes and progression to long-term opioid use is less understood. The present study aims to assess for an association between surgical approach in inguinal hernia repair and long-term opioid use. An a priori study protocol was prepared outlining the study design, variables and outcomes definition and the intended analysis. The intended analysis was to compare outcomes between surgical approaches (RAS, Lap, and Open) using logistic regression accounting for inverse probability of treatment weights (IPTW). The study followed the Strengthening the Reporting of Observational Studies in Epidemiology (STROBE) guidelines for reporting cohort studies, but it was not registered in a study repository.

## Materials and methods

### Study design

A retrospective cohort study was conducted using the Merative™ MarketScan® Research Databases, a large dataset which captures de-identified inpatient, outpatient and pharmacy service claims for over 273 million patients with employer-sponsored health insurance benefits [12]. The database includes de-identified healthcare information compliant to the Health Insurance Portability and Accountability Act (HIPAA), and as such the study was considered exempt from review by the institutional review board in accordance with 45 CFR §46.

**Fig. 1:**
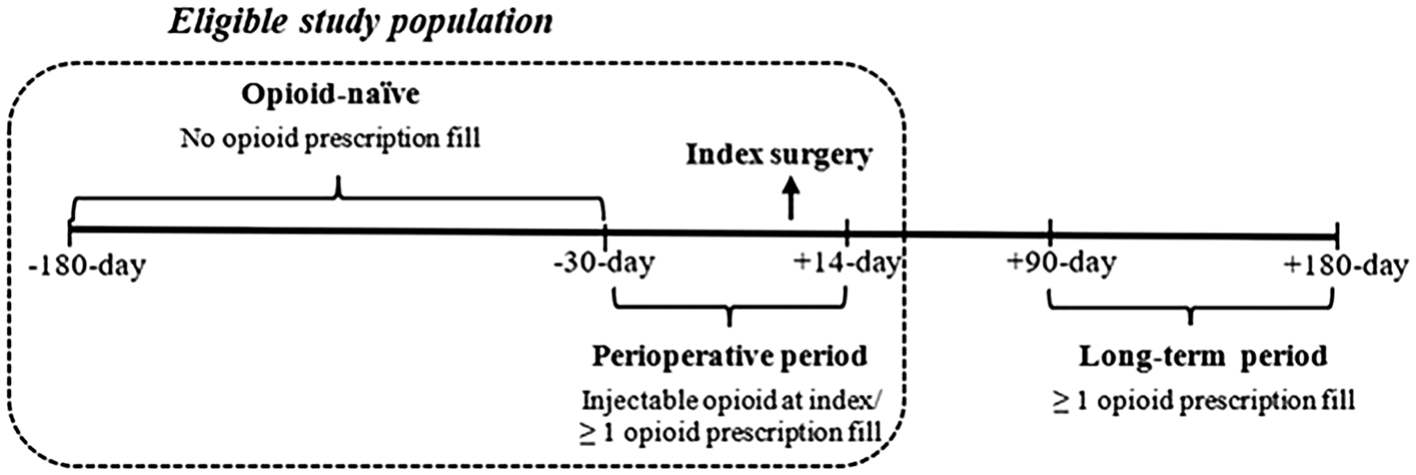
Study design

The study population was adults (≥ 18 years old) who underwent outpatient primary inguinal hernia repair between 2016 and 2020. As shown in Fig. 1, patients were required to be opioid-naïve (no opioid prescription between 30 and 180 days before surgery), and get exposed to opioid medication in the perioperative period (30 days before to 14 days after surgery). Consistent with prior studies, this was done to ensure only opioid-naïve patients who were exposed to opioids perioperatively are included [13, 14]. Exposure to opioid during the peri-operative period was identified with at least one opioid prescription fill from pharmacy claim, or procedure claim for injectable opioid during surgery. Patients were excluded if they had bilateral repair, were not continuously enrolled in medical and pharmacy insurance benefit, had malignancy, metastatic cancer, chronic pain, or opioid abuse/dependency diagnosis in the 180 days before surgery, or had invalid prescription fill information (zero/negative quantity supplied or days of supply). The International Classification of Diseases, Tenth Revision (ICD-10) codes, and the Current Procedural Terminology (CPT) codes shown in eTable 1 were used identify patient population, and differentiate surgical approaches (RAS, Lap, and Open).

### Outcome variables

The primary outcome for this study was long-term opioid use, defined as one or more opioid prescription filled between 90 and 180 days after surgery. This definition was consistent with prior studies, and a surrogate for post-surgical chronic pain per the International Association for Study of Pain 2019 definition [3, 13, 14]. Further, we assessed prescription fill for controlled substance schedule II or III opioids, per the Drug Enforcement Administration (DEA) classification, and high-dose opioid prescription fill, defined as over 50 morphine milligram equivalent (MME) opioid dose per day in the long-term. These definitions were considered to represent high-risk opioid use because of the potential of abuse with Schedule II/III opioids, and increased overdose risk with over 50 MME per day dosing with minimal additional benefit to the patient [15, 16].

### Study covariates

Patient characteristics assessed include age, sex, area-level income, region of residence, metropolitan residence status, type of health insurance plan, Charlson comorbidity score [17, 18], tobacco abuse and/or history, alcohol abuse and/or dependence, obesity/overweight status, mental health problem, and year of surgery. Types of health insurance plan were preferred provider organization (PPO), comprehensive insurance, health maintenance organization (HMO), point-of-service (POS) and other insurance plans.

### Statistical analysis

Descriptive statistical analysis of baseline patient characteristics was conducted by surgical approach. Outcomes were compared between RAS versus Lap, RAS versus open, and Lap versus open groups separately. To adjust for difference between groups and minimize selection bias, stabilized inverse probability of treatment weighting (IPTW) was performed when comparing outcomes for each cohort. Multivariable logistic regression including all baseline patient characteristics as covariates was used to generate propensity score for the calculation of IPTW. Adjustment with IPTW creates a synthetic sample which is independent of covariates and allows for estimation of unbiased average treatment effects [19]. To ensure no residual differences exist between groups, baseline characteristics were compared before and after IPTW adjustment using standardized mean/proportion difference, with < 0.1 difference considered comparable. All analyses were performed using R statistical software (version 4.2.2) [20]. A *p-*value of < 0.05 was considered statistically significant.

## Results

**Fig. 2:**
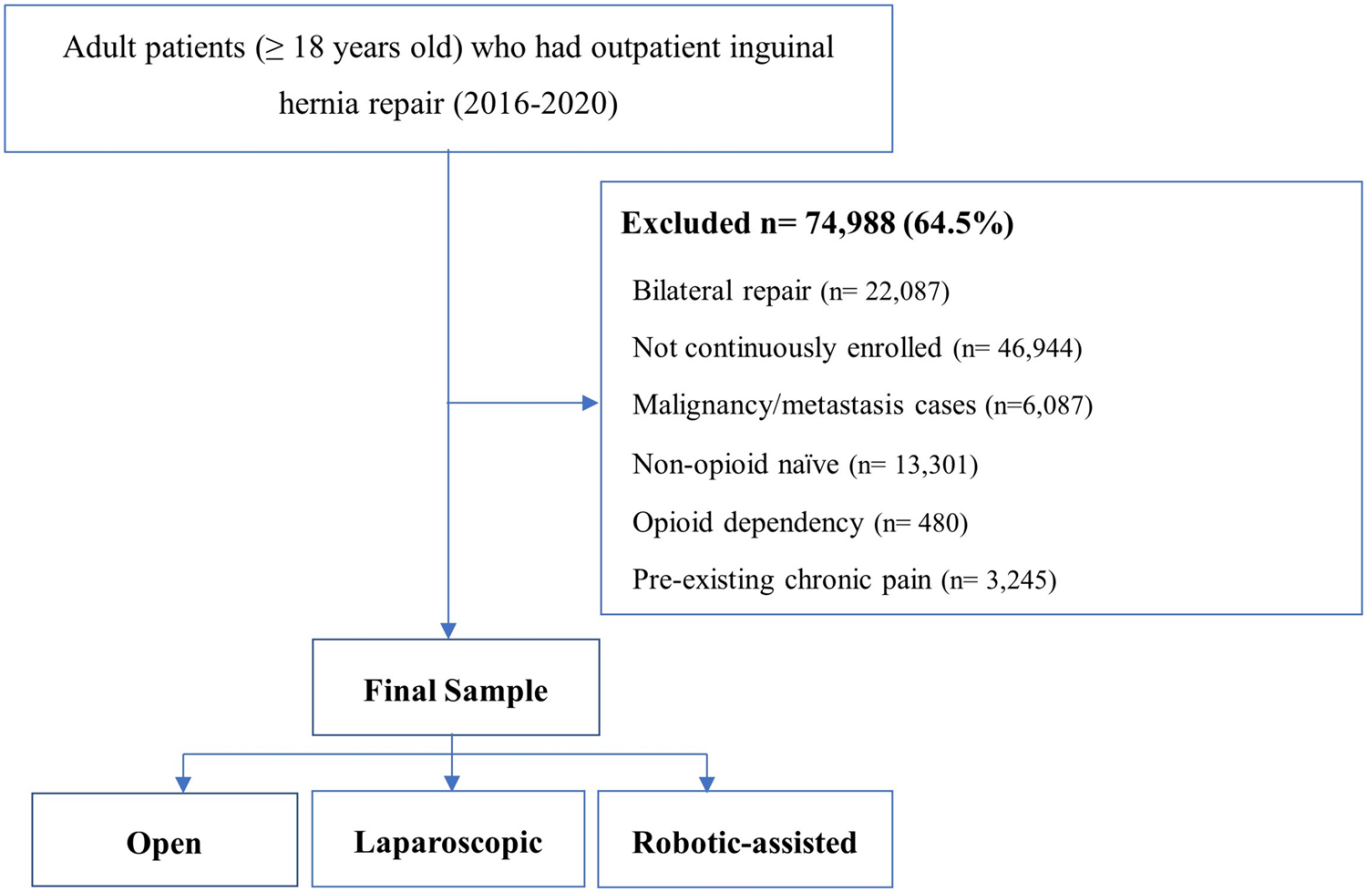
Sample selection flowchart

A total of 116,259 patients aged ≥ 18 years old who had outpatient primary inguinal hernia repair were identified from the Merative™ MarketScan® databases between 2016 and 2020. After applying exclusion criteria, 41,271 patients remained in the study sample, of whom 2070 (5.0%) had RAS, 16,704 (40.5%) Lap, and 22,497 (54.5%) open surgery (Fig. 2). As shown in Table 1, most of the sample were male (37,667 [91.3%]), had zero Charlson’s comorbidity score (332,619 [79.0%]), and over one-half of them had preferred payor organization health insurance plan (20,969 [50.8%]). Among open surgery group, there were greater proportion of ≥ 65 years old patients (*n* = 3427 [15.2%] versus RAS: *n* = 94 [4.5%], Std Diff. = − 0.364; and Lap: *n* = 1549 [9.3%], Std Diff. = 0.183), and greater proportion of patients with ≥ 2 Charlson comorbidity score (*n* = 1821 [8.1%] versus RAS: *n* = 108 [5.2%], Std Diff. = 0.108; and Lap: *n* = 901 [5.4%], Std Diff. = − 0.116). In contrast, there were greater proportion of obese/overweight patients in RAS (*n* = 261, [12.6%] versus Lap: *n* = 1538 [9.2%], Std Diff. = 0.109, and Open: *n* = 1939 [8.6%], Std Diff. = 0.130), and greater proportion of metropolitan residing patients (*n* = 1276 [83.4%] versus Lap: *n* = 13,069 [78.2%], Std Diff. = 0.131, and open: *n* = 16,799 [74.7%], Std Diff. = 0.215). After IPTW, all baseline characteristics were comparable (Std Diff. < 0.1) between surgical approaches (eTable 2).

**Table 1:** Sample baseline characteristics before inverse-probability of treatment weighting

Characteristic	Overall, *N* = 41,271	Open, *N* = 22,497	Lap, *N* = 16,704	RAS, *N* = 2070	Standardized difference		
					RAS vs lap	RAS vs open	Lap vs open
Age, *n* (%)							
18–44 years	11,117 (26.9)	5615 (25.0)	4867 (29.1)	635 (30.7)	0.034	0.128	− 0.094
45–54 years	10,069 (24.4)	5154 (22.9)	4359 (26.1)	556 (26.9)	0.017	0.092	− 0.074
55–64 years	15,015 (36.4)	8301 (36.9)	5929 (35.5)	785 (37.9)	0.050	0.021	0.029
65+	5070 (12.3)	3427 (15.2)	1549 (9.3)	94 (4.5)	− 0.187	− 0.364	0.183
Sex, male, *n* (%)	37,667 (91.3)	20,509 (91.2)	15,256 (91.3)	1902 (91.9)	0.020	0.026	− 0.006
Annual income, *n* (%)							
< $35,000	3370 (8.2)	1995 (8.9)	1238 (7.4)	137 (6.6)	− 0.031	− 0.084	0.053
$35,000–$40,000	11,824 (28.6)	6537 (29.1)	4696 (28.1)	591 (28.6)	0.010	− 0.011	0.021
$40,000+	16,819 (40.8)	9153 (40.7)	6962 (41.7)	704 (34.0)	− 0.159	− 0.138	− 0.020
Unknown	9258 (22.4)	4812 (21.4)	3808 (22.8)	638 (30.8)	0.182	0.216	− 0.034
Region, *n* (%)							
Northeast	7649 (18.5)	4597 (20.4)	2745 (16.4)	307 (14.8)	− 0.044	− 0.147	0.103
North Central	10,290 (24.9)	5546 (24.7)	4096 (24.5)	648 (31.3)	0.152	0.149	0.003
South	16,809 (40.7)	8812 (39.2)	7149 (42.8)	848 (41.0)	− 0.037	0.037	− 0.074
West	6402 (15.5)	3470 (15.4)	2669 (16.0)	263 (12.7)	− 0.094	− 0.078	− 0.015
Unknown	121 (0.3)	72 (0.3)	45 (0.3)	4 (0.2)	− 0.016	− 0.025	0.009
Metropolitan status, *n* (%)							
Metropolitan	31,594 (76.6)	16,799 (74.7)	13,069 (78.2)	1726 (83.4)	0.131	0.215	− 0.084
Non-metropolitan	5192 (12.6)	3183 (14.1)	1829 (10.9)	180 (8.7)	− 0.076	− 0.172	0.097
Unknown	4485 (10.9)	2515 (11.2)	1806 (10.8)	164 (7.9)	− 0.099	− 0.111	0.012
Insurance plan, *n* (%)							
PPO	20,969 (50.8)	11,473 (51.0)	8526 (51.0)	970 (46.9)	− 0.084	− 0.083	− 0.001
Comprehensive	2841 (6.9)	1857 (8.3)	862 (5.2)	122 (5.9)	0.032	− 0.092	0.124
HMO	4879 (11.8)	2692 (12.0)	1955 (11.7)	232 (11.2)	− 0.016	− 0.024	0.008
POS	2680 (6.5)	1497 (6.7)	1020 (6.1)	163 (7.9)	0.069	0.047	0.022
Others^a^	9277 (22.5)	4627 (20.6)	4098 (24.5)	552 (26.7)	0.049	0.144	− 0.095
Unknown	625 (1.5)	351 (1.6)	243 (1.5)	31 (1.5)	0.004	− 0.005	0.009
Charlson comorbidity, *n* (%)							
0	32,619 (79.0)	17,419 (77.4)	13,535 (81.0)	1665 (80.4)	− 0.015	0.074	− 0.089
1	5822 (14.1)	3257 (14.5)	2268 (13.6)	297 (14.3)	0.022	− 0.004	0.026
2+	2830 (6.9)	1821 (8.1)	901 (5.4)	108 (5.2)	− 0.008	− 0.116	0.108
Tobacco abuse/history, *n* (%)	3038 (7.4)	1705 (7.6)	1181 (7.1)	152 (7.3)	0.011	− 0.009	0.020
Obesity/overweight, *n* (%)	3738 (9.1)	1939 (8.6)	1538 (9.2)	261 (12.6)	0.109	0.130	− 0.021
Alcohol abuse/history, *n* (%)	325 (0.8)	196 (0.9)	118 (0.7)	11 (0.5)	− 0.022	− 0.041	0.019
Mental health problem (%)	3354 (8.1)	1782 (7.9)	1396 (8.4)	176 (8.5)	0.005	0.021	− 0.016
Year of surgery, *n* (%)							
2016	12,561 (30.4)	7678 (34.1)	4573 (27.4)	310 (15.0)	− 0.307	− 0.457	0.147
2017	10,364 (25.1)	5970 (26.5)	3999 (23.9)	395 (19.1)	− 0.118	− 0.178	0.060
2018	8527 (20.7)	4450 (19.8)	3610 (21.6)	467 (22.6)	0.023	0.068	− 0.045
2019	7321 (17.7)	3359 (14.9)	3289 (19.7)	673 (32.5)	0.295	0.422	− 0.126
2020	2498 (6.1)	1040 (4.6)	1233 (7.4)	225 (10.9)	0.121	0.235	− 0.116

*RAS* robotic-assisted surgery, *Lap* laparoscopic surgery, *Std Diff* standardized difference, *PPO* Preferred Payer Organization, *HMO* Health Maintenance Organization, *POS* point-of-service

^a^Others include basic/major medical benefits, exclusive provider organization, consumer driven health plan, and high deductible health plan

In IPTW adjusted analysis (Table 2), RAS was associated with 22% less likelihood of any opioid fill (OR 0.78, 95% CI 0.60 to 0.98, *p* = 0.041), and 26% less likelihood of schedule II/III opioid fill (OR 0.74, 95% CI 0.56 to 0.96, *p* = 0.029) in the long-term as compared to Lap. RAS and Lap were comparable in high-dose (> 50 MME per day) opioid fill (*p* = 0.840). As compared to Open, RAS was associated with 33% less likelihood of any opioid fill (OR 0.67, 95% CI 0.52 to 0.85, *p* = 0.002), and 32% less likelihood of schedule II/III opioid fill (OR 0.68, 95% CI 0.51 to 0.88, *p* = 0.004) in the long-term. RAS and Open were comparable in high-dose (> 50 MME per day) opioid fill (*p* = 0.860). There was no significantly different long-term opioid use between Lap versus Open in any opioid, schedule II/III opioid, and high-dose opioid fills (all *p* > 0.05).

**Table 2:** Inverse probability of treatment weighting adjusted comparison of long-term opioid use between surgical approaches

Opioid, *n* (%)	RAS versus lap				RAS versus open				Lap vs Open			
	Lap, *N* = 16,700	RAS, *N* = 2082	O.R. (95% CI)	*p*	Open, *N* = 22,495	RAS, *N* = 2066	O.R. (95% CI)	*p*	Open, *N* = 22,493	Lap, *N* = 16,712	O.R. (95% CI)	*p*
Any	762 (4.6)	75 (3.6)	0.78 (0.60–0.98)	0.041	1124 (5.0)	71 (3.4)	0.67 (0.52–0.85)	0.002	1109 (4.9)	781 (4.7)	0.95 (0.86–1.04)	0.250
Schedule II/III	649 (3.9)	61 (2.9)	0.74 (0.56–0.96)	0.029	944 (4.2)	60 (2.9)	0.68 (0.51–0.88)	0.004	933 (4.1)	659 (3.9)	0.95 (0.86–1.05)	0.310
High-dose^a^	134 (0.8)	16 (0.8)	0.95 (0.54–1.55)	0.840	198 (0.9)	17 (0.8)	0.96 (0.56–1.52)	0.860	196 (0.9)	139 (0.8)	0.96 (0.77–1.19)	0.710

All regression analyses were weighted by inverse probability of treatment weights (IPTW) estimated using logistic regression including baseline characteristics

*RAS* robotic-assisted surgery, *Lap* laparoscopic surgery, *MME* morphine milligram equivalent, *OR* odds ratio, *CI* confidence interval

^a^High-dose: Over 50 morphine milligram equivalent per day opioid fill

## Discussion

In this retrospective review of opioid-naïve adults undergoing outpatient primary unilateral inguinal hernia in the Merative™ MarketScan® database, long-term use of any opioid and schedule II/III opioid was significantly decreased in a robotic-assisted surgery approach as compared to both laparoscopic and open approaches, while there was no significant difference in these outcomes between the laparoscopic and open approaches. High-dose long-term opioid use was present in less than 1% of patients and did not vary between surgical approaches. In our results, the statistical significance and the effect magnitude for RAS is likely underestimated as it is known that administrative claims databases fail to capture all RAS operations, incorrectly categorizing many of them as Lap [21–23]. The overall effect sizes for any long-term opioid use in the RAS group were moderate with an odds ratio of 0.78 compared to lap and 0.67 compared to open. This effect can have a great impact when considering the scale at which this operation is performed. When the overall rate of progressing to long-term opioid use identified in our sample (4.8%) is extrapolated to the 800,000 inguinal hernia repairs performed annually in the United States [4], greater than 38,000 patients are progressing to long-term opioid use after inguinal hernia repair annually. In a population this large, a risk reduction of the magnitude identified in this study represents many thousands of patients.

Our findings are consistent with findings of recent similar studies. The percentage of patients proceeding to long-term opioid use is comparable to that described by in the literature for general surgery operations (6–10%) [3, 14]. A recent study by Howard et al. reported only 1.5% of patients developed new persistent opioid use after inguinal hernia repair, but the authors acknowledged that this may be an underestimate of the true rate [24]. The long-term opioid use results from this analysis expand on the existing body of evidence regarding opioid use after inguinal hernia repair. Multiple studies have concluded that although MIS inguinal hernia repair has a shorter recovery time and less acute pain [8], short-term opioid use does not differ by surgical approach [6, 10]. These studies generally have been limited by relatively small sample sizes. A study by Reinhorn et al. reported a significant difference in short-term opioid use after inguinal hernia repair by surgical approach, with less opioid use among the open surgery patients as compared to the MIS group [9]. This study compared MIS posterior (preperitoneal) mesh placement to open posterior mesh placement, the latter of which is an uncommonly performed technique. The body of evidence on long-term opioid use after inguinal hernia repair is more limited. Howard et al. found that new persistent opioid use after inguinal hernia repair was decreased in laparoscopic surgery as compared to open, with an odds ratio of 0.89 [24]. Robotic surgery was not assessed in that study.

The mechanism by which RAS may reduce long-term opioid use compared to lap and open surgery has several potential explanations. The first and most intuitive possibility is that RAS produces less long-term pain. This explanation is especially plausible comparing RAS to open surgery. Though the quality of evidence is low, prior studies suggest that rates of developing chronic pain after inguinal hernia repair are greater for open repair than for MIS repair [7, 8]. No such difference in chronic pain rates has been demonstrated between RAS and lap approaches. Many benefits for RAS over lap surgery for inguinal hernia repair have been anecdotally described but are lacking in higher level evidence. These include improved three-dimensional visualization, minimization of tissue trauma, and improved visualization and protection of retroperitoneal neurovascular structures. The effect associated with RAS may also be due to differences in operative technique between RAS and lap approaches. For example, it may be more common to close the peritoneal flap with suture during RAS, while penetrating tacks may be more commonly used for this purpose in lap surgery. Tacks and other penetrating fixation methods increase the risk of injury to the ilioinguinal or iliohypogastic nerves as they traverse between muscle layers of the lateral abdominal wall, and the use of permanent tacks has been identified as a risk factor for requesting opioid prescription refill following inguinal hernia repair [5]. The heterogeneity in surgical technique, even within a given surgical approach, leaves correlating technical aspects of surgery to outcomes in the realm of speculation.

Further, there is no certainty that the difference in long-term opioid use is directly due to surgical approach or resultant amount of pain. Multiple studies have demonstrated that opioid use is associated with prescriber practice, with use increasing with larger number of MME prescribed following the operation [5, 11]. Prescription size following surgery has also been associated with increased prescription refill requests and new persistent opioid use after inguinal hernia repair [5, 24]. It is possible that surgeons who are early-adopters of RAS may also be early-adopters of certain opioid prescribing or post-operative care practices that minimize long-term opioid use. Other potential confounding variables include the specifics of patient and approach selection by individual surgeons, which could not be measured in this sample.

This study is limited by its reliance on claims data and the known limits in accuracy of these data, including the under capturing of RAS as described above. It is similarly unknown the reasons of opioid fill and how many patients who had a long-term opioid prescription fill progressed to become regular opioid users or fell victim to long-term opioid abuse or addiction. As such, generalizations about the contribution of these patterns to the overall opioid epidemic must be made with caution.

## Conclusion

Overall, this analysis provides an important insight into the overall risk of progressing to long-term opioid use following inguinal hernia repair, and suggests that patients undergoing RAS has a decreased risk of long-term opioid use compared to those undergoing lap and open. This information may aid surgeons in selecting the optimal surgical approach for inguinal hernia repair, considering not only anesthetic and operative risk, but also risk of long-term opioid use. It remains in patients’ best interests for surgeons to be competent in a range of options for inguinal hernia repair in order to provide optimal personalized recommendations.

### Supplementary Information

Below is the link to the electronic supplementary material.

Supplementary file1 (DOCX 22 kb)
