# Supplementary material for: New persistent opioid use following robotic-assisted, laparoscopic and open surgery inguinal hernia repair
Source: Surg Endosc. 2024 Jul 22;38(9):5153–9. doi: 10.1007/s00464-024-11040-1 (PMC11362387; doi:10.1007/s00464-024-11040-1)
Supplement: Supplementary file 1 — Supplementary file1 (DOCX 22 kb) [file 464_2024_11040_MOESM1_ESM.docx]

eTable 1. Diagnosis and Procedure codes used

| Variables | Diagnosis (Dx) and procedure (Proc) Codes by Type | |
| --- | --- | --- |
|  | ICD-10 | CPT/HCPCS |
| Initial inguinal hernia repair (Proc) |  |  |
| Open | (Right IHR) 0YQ50ZZ, 0YU507Z, 0YU50JZ, 0YU50KZ (Left IHR) 0YQ60ZZ, 0YU607Z, 0YU60JZ, 0YU60KZ | 49505, 49507 (Right or Left IHR) |
| Laparoscopic | (Right IHR) 0YQ53ZZ, 0YQ54ZZ, 0YU547Z, 0YU54JZ, 0YU54KZ  (Left IHR) 0YQ63ZZ, 0YQ64ZZ, 0YU647Z, 0YU64JZ, 0YU64KZ | 49650 (Right or Left IHR) |
| Robotic-assisted^1^ | *8E0W0CZ, 8E0W3CZ, 8E0W4CZ,* *8E0W7CZ, 8E0W8CZ, 8E0WXCZ* | *S2900* |
| Pre-index chronic pain (Dx) | G8921, G8922, G8928, G8929 | - |
| Opioid abuse/dependency (Dx) | F11.xxx |  |
| Obesity/overweight (Dx) | Z68.3x, Z68.4x, E66.xx | - |
| Mental health problem (Dx) | F20.xx, F31.xx, F32.0, F32.1, F32.2, F32.3, F32.4, F32.5, F32.89, F32.9, F33.xx, F34.0, F34.1, F41.x, F42.x, F44.xx, F45.xx, F48.1, F48.2, F48.9, F68.1x, F68.8 | - |
| Tobacco abuse/history (Dx) | F17.2xx, Z87.891 | - |
| Alcohol abuse/history (Dx) | F10.1xx, F10.2xx |  |
| Injectable Opioids | - | J0595, J0745, J1810, J3010, J1170, J0364, J2270, J2271, J2274, J2275, J1960, J2175, J2180, J1230, J2410, J3070 |

**Abbreviations**: Dx, Diagnosis; Proc, Procedure; IHR, Inguinal Hernia Repair

^1^ These codes were used as modifier codes for robotic-assisted surgery (RAS) in the presence of a laparoscopic or open IHR code.

Patients who had both “Right IHR” and “Left IHR” codes, or had procedure modifier codes for bilateral IHR (“50”, or both “LT” and “RT”) were considered to have undergone bilateral IHR.

eTable 2. Baseline Characteristics after Inverse-probability of Treatment Weighting Adjustment

| **Characteristic** | **RAS vs Lap** | | |  | **RAS vs Open** | | |  | **Lap vs Open** | | |
| --- | --- | --- | --- | --- | --- | --- | --- | --- | --- | --- | --- |
|  | **Lap**,  N = 16,700 | **RAS**,  N = 2,082 | **Std Diff** |  | **Open**,  N = 22,495 | **RAS**,  N = 2,066 | **Std Diff** |  | **Open**,  N = 22,493 | **Lap,** N = 16,712 | **Std Diff** |
| **Age, n (%)** |  |  |  |  |  |  |  |  |  |  |  |
| 18-44 years | 4,894 (29.3) | 609 (29.2) | -0.001 |  | 5,722 (25.4) | 533 (25.8) | 0.008 |  | 6,020 (26.8) | 4,471 (26.8) | 0.000 |
| 45-54 years | 4,373 (26.2) | 550 (26.4) | 0.005 |  | 5,231 (23.3) | 490 (23.7) | 0.010 |  | 5,451 (24.2) | 4,049 (24.2) | 0.000 |
| 55-64 years | 5,971 (35.8) | 743 (35.7) | -0.002 |  | 8,319 (37.0) | 777 (37.6) | 0.013 |  | 8,169 (36.3) | 6,075 (36.4) | -0.001 |
| 65+ | 1,462 (8.8) | 180 (8.7) | -0.004 |  | 3,223 (14.3) | 267 (12.9) | -0.048 |  | 2,852 (12.7) | 2,116 (12.7) | 0.001 |
| **Sex, Male, n (%)** | 15,263 (91.4) | 1,902 (91.4) | -0.001 |  | 20,522 (91.2) | 1,895 (91.7) | 0.017 |  | 20,524 (91.2) | 15,246 (91.2) | 0.001 |
| **Annual income, n (%)** |  |  |  |  |  |  |  |  |  |  |  |
| < $35,000 | 1,224 (7.3) | 159 (7.6) | 0.012 |  | 1,954 (8.7) | 195 (9.5) | 0.029 |  | 1,865 (8.3) | 1,393 (8.3) | -0.002 |
| $35,000 - $40,000 | 4,703 (28.2) | 578 (27.8) | -0.008 |  | 6,526 (29.0) | 579 (28.0) | -0.021 |  | 6,438 (28.6) | 4,784 (28.6) | 0.000 |
| $40,000 + | 6,825 (40.9) | 868 (41.7) | 0.017 |  | 9,032 (40.2) | 853 (41.3) | 0.023 |  | 9,231 (41.0) | 6,838 (40.9) | 0.003 |
| Unknown | 3,948 (23.6) | 477 (22.9) | -0.017 |  | 4,983 (22.2) | 439 (21.2) | -0.021 |  | 4,959 (22.0) | 3,696 (22.1) | -0.002 |
| **Region, n (%)** |  |  |  |  |  |  |  |  |  |  |  |
| Northeast | 2,718 (16.3) | 359 (17.2) | 0.026 |  | 4,493 (20.0) | 458 (22.2) | 0.058 |  | 4,218 (18.8) | 3,142 (18.8) | -0.001 |
| North Central | 4,209 (25.2) | 492 (23.6) | -0.036 |  | 5,664 (25.2) | 474 (22.9) | -0.050 |  | 5,525 (24.6) | 4,096 (24.5) | 0.001 |
| South | 7,120 (42.6) | 905 (43.5) | 0.017 |  | 8,850 (39.3) | 818 (39.6) | 0.005 |  | 9,162 (40.7) | 6,807 (40.7) | 0.000 |
| West | 2,609 (15.6) | 322 (15.4) | -0.005 |  | 3,419 (15.2) | 311 (15.1) | -0.004 |  | 3,520 (15.6) | 2,615 (15.6) | 0.000 |
| Unknown | 44 (0.3) | 5 (0.2) | -0.008 |  | 70 (0.3) | 5 (0.2) | -0.016 |  | 68 (0.3) | 51 (0.3) | -0.001 |
| **Metropolitan status, n (%)** |  |  |  |  |  |  |  |  |  |  |  |
| Metropolitan | 13,160 (78.8) | 1,642 (78.9) | 0.003 |  | 16,962 (75.4) | 1,560 (75.5) | 0.003 |  | 17,119 (76.1) | 12,699 (76.0) | 0.003 |
| Non-metropolitan | 1,788 (10.7) | 223 (10.7) | 0.001 |  | 3,080 (13.7) | 285 (13.8) | 0.003 |  | 2,888 (12.8) | 2,166 (13.0) | -0.004 |
| Unknown | 1,752 (10.5) | 216 (10.4) | -0.004 |  | 2,453 (10.9) | 221 (10.7) | -0.007 |  | 2,486 (11.1) | 1,847 (11.1) | 0.000 |
| **Insurance plan, n (%)** |  |  |  |  |  |  |  |  |  |  |  |
| PPO | 8,448 (50.6) | 1,045 (50.2) | -0.008 |  | 11,393 (50.6) | 1,034 (50.1) | -0.012 |  | 11,477 (51.0) | 8,536 (51.1) | -0.001 |
| Comprehensive | 870 (5.2) | 89 (4.3) | -0.042 |  | 1,808 (8.0) | 123 (5.9) | -0.082 |  | 1,558 (6.9) | 1,155 (6.9) | 0.001 |
| HMO | 1,946 (11.7) | 252 (12.1) | 0.014 |  | 2,679 (11.9) | 264 (12.8) | 0.028 |  | 2,661 (11.8) | 1,973 (11.8) | 0.001 |
| POS | 1,056 (6.3) | 144 (6.9) | 0.024 |  | 1,524 (6.8) | 167 (8.1) | 0.051 |  | 1,445 (6.4) | 1,075 (6.4) | 0.000 |
| Others^1^ | 4,137 (24.8) | 521 (25.0) | 0.006 |  | 4,742 (21.1) | 446 (21.6) | 0.012 |  | 5,011 (22.3) | 3,720 (22.3) | 0.000 |
| Unknown | 244 (1.5) | 31 (1.5) | 0.003 |  | 350 (1.6) | 31 (1.5) | -0.003 |  | 340 (1.5) | 252 (1.5) | 0.000 |
| **Charlson comorbidity score, n (%)** |  |  |  |  |  |  |  |  |  |  |  |
| 0 | 13,519 (81.0) | 1,686 (81.0) | 0.001 |  | 17,473 (77.7) | 1,616 (78.2) | 0.013 |  | 17,759 (79.0) | 13,192 (78.9) | 0.000 |
| 1 | 2,283 (13.7) | 284 (13.7) | -0.001 |  | 3,256 (14.5) | 295 (14.3) | -0.006 |  | 3,168 (14.1) | 2,352 (14.1) | 0.000 |
| 2 + | 898 (5.4) | 112 (5.4) | 0.000 |  | 1,767 (7.9) | 156 (7.5) | -0.013 |  | 1,566 (7.0) | 1,167 (7.0) | -0.001 |
| **Tobacco abuse/history, n (%)** | 1,185 (7.1) | 147 (7.0) | -0.002 |  | 1,700 (7.6) | 162 (7.9) | 0.011 |  | 1,657 (7.4) | 1,234 (7.4) | -0.001 |
| **Obesity/overweight, n (%)** | 1,597 (9.6) | 192 (9.2) | -0.011 |  | 2,013 (9.0) | 182 (8.8) | -0.005 |  | 1,995 (8.9) | 1,486 (8.9) | -0.001 |
| **Alcohol abuse/history, n (%)** | 115 (0.7) | 13 (0.6) | -0.009 |  | 189 (0.8) | 18 (0.9) | 0.003 |  | 181 (0.8) | 135 (0.8) | 0.000 |
| **Mental health problem (%)** | 1,398 (8.4) | 164 (7.9) | -0.017 |  | 1,793 (8.0) | 154 (7.5) | -0.019 |  | 1,824 (8.1) | 1,353 (8.1) | 0.001 |
| **Year of surgery, n (%)** |  |  |  |  |  |  |  |  |  |  |  |
| 2016 | 4,344 (26.0) | 534 (25.6) | -0.009 |  | 7,315 (32.5) | 647 (31.3) | -0.028 |  | 7,036 (31.3) | 5,232 (31.3) | -0.001 |
| 2017 | 3,910 (23.4) | 490 (23.6) | 0.004 |  | 5,829 (25.9) | 549 (26.6) | 0.016 |  | 5,726 (25.5) | 4,261 (25.5) | -0.001 |
| 2018 | 3,625 (21.7) | 445 (21.4) | -0.008 |  | 4,500 (20.0) | 404 (19.5) | -0.011 |  | 4,610 (20.5) | 3,415 (20.4) | 0.002 |
| 2019 | 3,522 (21.1) | 443 (21.3) | 0.004 |  | 3,693 (16.4) | 352 (17.0) | 0.015 |  | 3,815 (17.0) | 1,259 (18.1) | 0.002 |
| 2020 | 1,299 (7.8) | 170 (8.2) | 0.013 |  | 1,159 (5.2) | 114 (5.5) | 0.014 |  | 1,305 (5.8) | 1,128 (16.3) | 0.006 |

**Abbreviations**: RAS, Robotic-assisted surgery; Lap, Laparoscopic Surgery; Std Diff, Standardized Difference; PPO, Preferred Payer Organization; HMO, Health Maintenance Organization; POS, Point of Service

^1^ Others include basic/major medical benefits, exclusive provider organization, consumer driven health plan, and high deductible health plan.
